# Novel chloroquine derivative suppresses melanoma cell growth by DNA damage through increasing ROS levels

**DOI:** 10.1111/jcmm.17260

**Published:** 2022-03-25

**Authors:** Jiaoduan Li, Jing Long, Jianglin Zhang, Nian Liu, Bei Yan, Ling Tang, Xiang Chen, Cong Peng

**Affiliations:** ^1^ Department of Dermatology Xiangya Hospital, Central South University Changsha China; ^2^ Hunan Key Laboratory of Skin Cancer and Psoriasis Xiangya Hospital Changsha China; ^3^ Hunan Engineering Research Center of Skin Health and Disease Xiangya Hospital Changsha China; ^4^ National Clinical Research Center for Geriatric Disorders Xiangya Hospital, Central South University Changsha China; ^5^ Department of Dermatology, Shenzhen People’s Hospital, The Second Clinical Medical College, Jinan University, The First Affiliated Hospital Southern University of Science and Technology Shenzhen China

**Keywords:** chloroquine derivative, DNA damage, G2/M arrest and apoptosis, melanoma, ROS

## Abstract

Melanoma is a fatal cancer with a significant feature of resistance to traditional chemotherapeutic drugs and radiotherapy. A mutation in the kinase BRAF is observed in more than 66% of metastatic melanoma cases. Therefore, there is an urgent need to develop new BRAF‐mutant melanoma inhibitors. High‐dose chloroquine has been reported to have antitumour effects, but it often induces dose‐limiting toxicity. In this study, a series of chloroquine derivatives were synthesized, and lj‐2‐66 had the best activity and was selected for further investigation. Furthermore, the anti‐BRAF‐mutant melanoma effect and mechanism of this compound were explored. CCK‐8 and colony formation assays indicated that lj‐2‐66 significantly inhibited the proliferation of BRAF‐mutant melanoma cells. Flow cytometry revealed that lj‐2‐66 induced G2/M arrest in melanoma cells and promoted apoptosis. Furthermore, lj‐2‐66 increased the level of ROS in melanoma cells and induced DNA damage. Interestingly, lj‐2‐66 also played a similar role in BRAF inhibitor‐resistant melanoma cells. In summary, we found a novel chloroquine derivative, lj‐2‐66, that increased the level of ROS in melanoma cells and induced DNA damage, thus leading to G2/M arrest and apoptosis. These findings indicated that lj‐2‐66 may become a potential therapeutic drug for melanoma harbouring BRAF mutations.

## 1 INTRODUCTION

Melanoma is a solid tumour resulting from malignant transformation of melanocytes in the skin and other organs. Although melanoma is rare (accounting for only 4% of skin cancer cases), it is a very deadly disease that accounts for 75% of skin cancer deaths.^1^ BRAF mutation occurs in more than 66% of cases of metastatic melanoma.^2^ The discovery of BRAF inhibitors such as vemurafenib and dabrafenib has improved the prognosis of melanoma patients.^3^ However, a large proportion of patients rapidly develop secondary drug resistance.^4^ Therefore, it is an urgent task to develop new drugs for melanomas with BRAF mutations.

Chloroquine has been found to exert inhibitory effects against melanoma via a variety of mechanisms. For example, it promotes the apoptosis of melanoma cells by inhibiting the degradation of the P53 upregulated modulator of apoptosis (PUMA) protein, suppresses melanoma cell invasion and metastasis by normalizing the tumour vasculature, and increases the sensitivity of GNAQ/11‐mutant melanoma to MEK1/2 inhibition.^5^, ^6^, ^7^ However, although chloroquine has considerable antimelanoma effects, a very high dose is often required to achieve these antitumour effects due to its weak antitumour activity. Systemic application of high‐dose chloroquine may cause extensive and serious side effects, among which the most serious complications are retinopathy, cardiomyopathy, neuromuscular disease and myopathy.^8^, ^9^

Chloroquine derivatives containing a 7‐chloroquinoline moiety were reported to have high antitumour activity.^10^, ^11^, ^12^, ^13^, ^14^ Moreover, 2‐methylquinoline and methylcarbazole were found to be important fragments with antitumour activity.^15^, ^16^, ^17^ In our previous report, we found that the 2‐methylquinoline and carbazole hybrid compounds 5I and 8g have certain antimelanoma activity.^18^ Based on the above information, we speculated that combining 2‐methyl‐7‐chloroquinoline and methylcarbazole hybrid fragments may be a promising direction for the design of inhibitors targeting BRAF‐mutant melanoma. Thus, we designed and synthesized a variety of chloroquine derivatives—2‐methyl‐7‐chloroquinoline and methylcarbazole hybrid compounds (Table 1 and Figure S1). Among these compounds, lj‐2‐66, containing a 2‐methyl‐7‐chloroquinoline fragment and a methylcarbazole fragment connected by a carbon atom linker, had the highest anti‐BRAF‐mutant melanoma activity.

**TABLE 1:** Chloroquine derivatives had antimelanoma activity

Compound		IC50(μM)in Sk‐Mel−5 cell growth inhibition*
lj‐2‐64	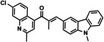	11.84
lj‐2‐65	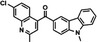	6.79
lj‐2‐66	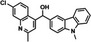	0.13
lj‐2‐67	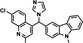	5.66
lj‐2‐68	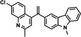	2.73
lj‐2‐114	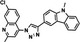	4.352

* Data are presented as mean ± SD, *n* = 3, from three independent experiments.

Through further study, we found that lj‐2‐66 exerts antitumour effects in melanoma harboring BRAF mutations both in vivo and in vitro and that the effective concentration was much lower than that of chloroquine. Further research showed that the compound causes DNA damage by increasing reactive oxygen species (ROS) levels, leading to G2/M arrest and apoptosis. In addition, lj‐2‐66 suppressed the proliferation of BRAF inhibitor‐resistant (BRAFi‐resistant) melanoma cells independent of their resistance status, suggesting that lj‐2‐66 may be a potential drug for BRAF‐mutant melanoma treatment.

## 2 MATERIALS AND METHODS

### 2.1 Chemicals

The method for synthesizing chloroquine derivatives is outlined in Figure S1. Cross‐dehydrogenative coupling of 1 with aldehyde 2 using hypervalent iodine and a TMSN_3_ system afforded Compound 3, which was then condensed with 4 to produce the product lj‐2‐64. By changing aldehydes 2 to 4 using the same strategy described for the synthesis of Compound 3, we easily obtained lj‐2‐65. With the important intermediate lj‐2‐65 in hand, we could easily prepare lj‐2‐66, lj‐2‐67 and lj‐2‐68 by reduction with NaBH4, substitution with N,N‐carbonyldiimidazole (CDI), and Wittig olefination with methyltriphenylphosphonium bromide (Compound 5) respectively. Then, lj‐2‐114 was prepared by modified click chemistry by condensation of 8 and 7 using a CuSO_4_/sodium L‐ascorbate/Et_3_N system, and 7 was obtained by substitution of 6 with NaN_3_.

### 2.2 Cell culture

The human melanoma cell lines Sk‐Mel‐5, Sk‐Mel‐28 and A375 (maintained in our laboratory) and the human melanocyte cell line PIG1 (a gift from Department of Dermatology, Xiangya Third Hospital) were used in this study. Sk‐Mel‐5, Sk‐Mel‐28 and A375 cells were cultured in Dulbecco’s modified Eagle’s medium (BI, Israel) supplemented with 10% foetal bovine serum (FBS) (BI, Israel) at 37 °C in 5% CO_2_. PIG1 was cultured in Medium 254 (Gibco) supplemented with 10% FBS (BI, Israel) at 37°C in 5% CO_2_. A375 cells were continually treated with 2 µM vemurafenib for more than 3 months to obtain a vemurafenib‐resistant cell line (labelled RA), and the drug was removed one week before use.

### 2.3 Cytotoxicity assay

Cells were seeded into 96‐well plates (1.5 × 10^3^ cells in each well), cultured in medium containing 10% FBS and incubated overnight at 37°C in 5% CO_2_. Then, different concentrations of lj‐2‐66 or dimethyl sulfoxide (DMSO) (control group) were added to the wells for 24, 48 or 72 h. Then, a Cell Counting Kit‐8 (CCK‐8) (Selleck, USA) was used to detect cell viability according to the instructions for use. A spectrophotometer (Beckman, USA) was used to detect the fluorescence of each well at a wavelength of 450 nm. GraphPad software was used to calculate the half‐maximal inhibitory concentration (IC_50_) after 48 h of treatment.

### 2.4 Colony formation assay

Cells were seeded into 6‐well plates (1.5 × 10^3^ cells in each well), cultured in complete growth medium, and incubated overnight at 37°C in 5% CO_2_. The next day, different concentrations of lj‐2‐66 or DMSO (control group) were added to the wells. After 48 h, the drug‐containing medium was replaced with medium containing 10% FBS. Culture was terminated after colonies were macroscopically visible (approximately 2–3 weeks). Then, the colonies were washed twice with phosphate buffered saline (PBS), fixed with 4% paraformaldehyde for 15 min and stained with 0.5% crystal violet staining solution for 20 min. After counting the colonies, GraphPad software was used for statistical analysis of the data.

### 2.5 Apoptosis assay

Cells were seeded into six‐well plates at a density of 3 × 10^5^ cells per well, incubated overnight in complete growth medium at 37°C and treated with different concentrations of lj‐2‐66 or DMSO (control group). After 48 h of treatment, the cells were washed twice with precooled PBS and digested with ethylene diamine tetraacetic acid‐free (EDTA‐free) trypsin digestion solution. Then, the collected cells were washed with PBS and incubated with Annexin V/propidium iodide stain (Biyuntian, C1062, China) according to the instruction manual. Apoptosis was detected by flow cytometry, and data were analysed with FlowJo software. Each sample was analysed in triplicate.

### 2.6 Cell cycle assay

Cells were seeded into six‐well plates at a density of 3 × 10^5^ cells per well, incubated overnight in complete growth medium at 37°C and treated with different concentrations of lj‐2‐66 or DMSO (control group). After 48 h, the cells were washed twice with precooled PBS and digested with 0.25% trypsin digestion solution. The collected cells were fixed with pre‐cooled 70% ethanol and incubated overnight at 4°C. Then, the collected cells were incubated with propidium iodide stain (Biyuntian, C1052, China) according to the instruction manual. The cell cycle assay was conducted by flow cytometry, and data were analysed with ModFit software. Each sample was analysed in triplicate.

### 2.7 Immunoblotting

Cells were lysed with RIPA lysis buffer containing protease and phosphatase inhibitors (Selleck, USA), and protein quantification was performed with BCA according to the instruction manual. Subsequently, appropriate amounts of proteins were prepared for Western blot analysis. Proteins were separated by 10%–12% SDS–PAGE and transferred to PVDF membranes (Millipore, MA, USA). Then, the membranes were incubated with antibodies specific for PARP (1:1000; CST; 9532S), P21 (1:1000; CST; 2947S), P53 (1:500; Santa Cruz; 47698), p‐P53 (1:1000; CST), BAX (1:5000; Proteintech; 50599), phospho‐histone H2AX (γH2AX) (1:1000; CST; 9718S) and GAPDH (1:5000; Proteintech; 60004‐1‐lg). Finally, the membranes were imaged with an image analysis system (Bio–Rad, USA).

### 2.8 RNA sequencing (RNA‐seq)

After treating Sk‐Mel‐28 cells with 100nM lj‐2‐66 or an equal volume of DMSO (control group) for 24 or 48 h, the cells were collected and sent to Wuhan Huada Sequencing Company for RNA‐seq.

### 2.9 Quantitative real‐time PCR analysis

Total RNA was extracted with TRIzol reagent (Invitrogen, USA). 2 µg of RNA was used as a template for the reverse transcription reaction. The primer sequences are as follows: hCDKN1AFw5′gcccgtgagcgatggaacttc3′; hCDKN1ARv5′cctgcctcctcccaactcatcc3′; hGADD45AFw5′ctggagagcagaagaccgaaagc3′; hGADD45ARv5′acatctctgtcgtcgtcctcgtc3′; hBBC3Fw5′tacgagcggcggagacaagag3′; hBBC3Rv5′ggcaggagtcccatgatgagattg3′; hCDK1Fw5′acaggtcaagtggtagccatga3′; hCDK1Rv5′gcataagcacatcctgaagactgac3′; hMCM3Fw5′accagaccatcaccatccaggag3′; hMCM3Rv5′aggcttcgctttatccaccaagtc3′; hMCM4Fw5′cctcgcctggagtggacctg3′; hMCM4Rv5′gagtgccgtatgtcagtggtgaac3′; hMCM5Fw5′tggactgacagcctcggtgatg3′; hMCM5Rv5′ggattgccacacggtcatcttctc3′; hMCM6Fw5′cctgcctaccagacacaagattcg3′; hMCM6Rv5′gcacagaaaagttccgctcacaag3′.

### 2.10 Measurement of ROS

Cells were treated with 100 nM lj‐2‐66 or an equal volume of DMSO (control group) for 3 or 6 h. In another experiment, cells were pretreated with 5 mmol/L N‐acetyl‐L‐cysteine (NAC) for 1 h before exposure to 100 nM lj‐2‐66 or an equal volume of DMSO (control group) for 6 h. Then, the cells were collected and incubated with 2,7‐dichlorodihydrofluorescein diacetate (DCFH‐DA) (Solarbio, China) for 20 min. ROS levels were detected by flow cytometry and analysed with FlowJo software.

### 2.11 Immunofluorescence

Cells (2 × 10^4^ cells/well) were seeded on coverslips in medium containing 10% FBS, incubated overnight at 37°C and exposed to different concentrations of lj‐2‐66 or DMSO (control group). After 24 h, the cells were digested with 0.25% trypsin digestion solution and incubated with an anti‐phospho‐histone H2AX mAb (1:1000; CST; 9718S) at 4°C overnight. Then, the cells were incubated with secondary antibodies at room temperature for 1 h and counterstained with DAPI. Images were acquired by confocal microscopy (Leica SP8).

### 2.12 Xenograft tumour model

The animal study was approved by the Ethics Committee of Central South University (China), strictly adhering to the “3R” principle of experimental animals. Sk‐Mel‐5 cells (2 × 10^6^) were subcutaneously injected into the right flanks of female BALB/c nude mice (5 weeks old). When the tumours reached approximately 50 mm^3^, 3 mg/kg lj‐2‐66 or an equal volume of corn oil (control group) was injected intraperitoneally every day. Tumour volumes and mouse body weights were recorded every other day. Each group contained 6 mice, and the tumour volume was calculated with the Formula *V* = 1/2(length × width^2^). When the volume of the largest tumour reached 1000 mm^3^, the tumours were collected and photographed.

### 2.13 Immunohistochemistry

Mouse tumours were fixed with formalin and embedded in paraffin. The slices were placed in a 65°C oven for 1.5 h. After the tissue was dewaxed with turpentine, an alcohol gradient was used to rehydrate the tissue. The slices were placed in preboiled sodium citrate and incubated in a pressure cooker for 6 min for antigen retrieval. After the sections returned to room temperature, they were blocked with peroxidase for 10 min and with goat serum for 1 h. Then, the slides were incubated with primary antibodies specific for Ki67 (1:300, Abcam, ab16667), P21 (1:50; CST; 2947S) and P53 (1:50; Santa Cruz; 47698) in the dark at 4 °C overnight. The next day, the slides were incubated with the secondary antibody and stained with 3,3′‐diaminobenzidine (DAB).

### 2.14 Statistical analysis

The statistical results are presented as the mean ± SEM values and were analysed by Student’s *t* test and one‐ or two‐way ANOVA with GraphPad Prism software (version 6.01). A *p* value <.05 was considered to be significant.

## 3 RESULTS

### 3.1 Chloroquine derivatives had antimelanoma activity

**FIGURE 1:**
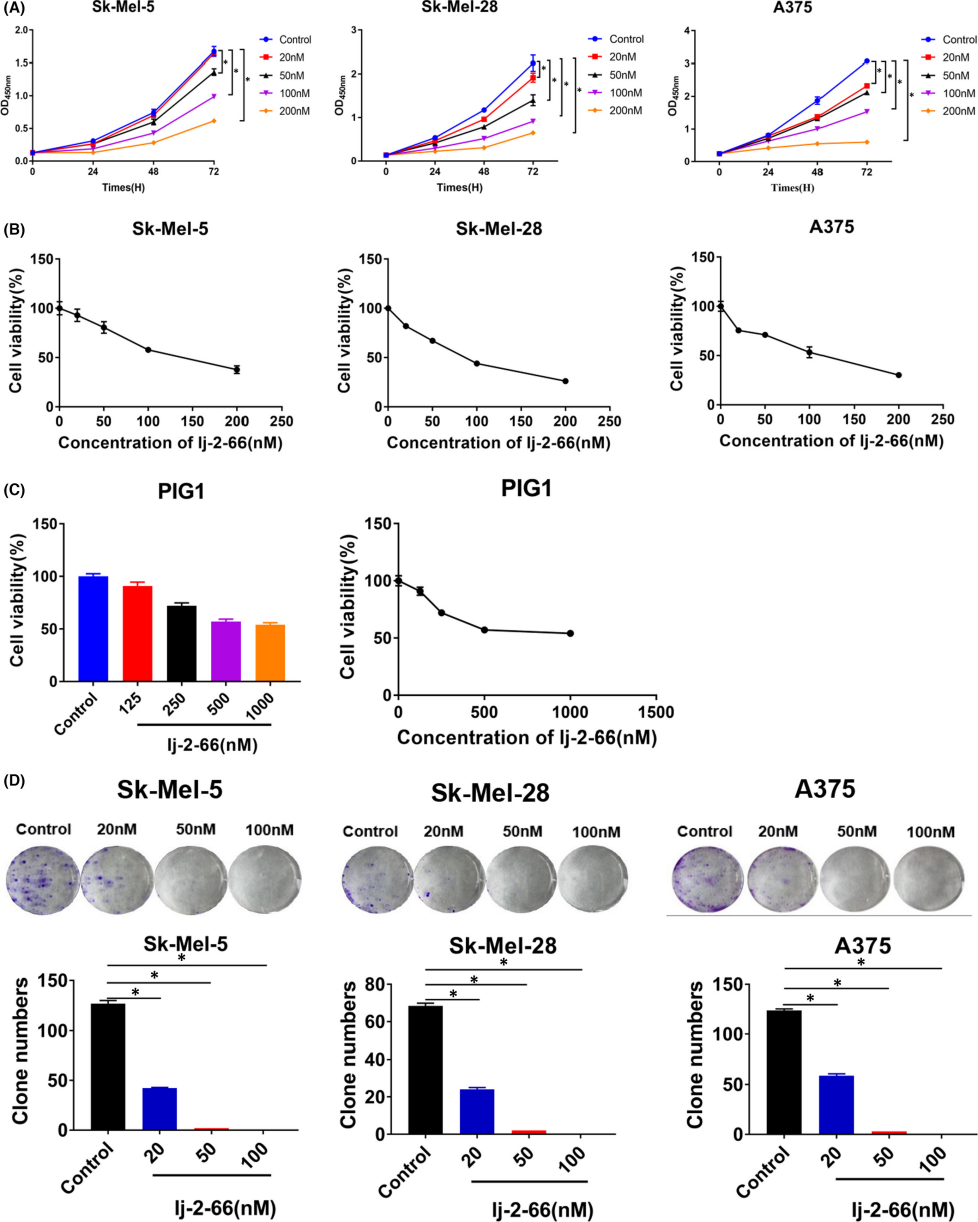
Effects of lj‐2‐66 on the proliferation of melanoma cells. (a) SK‐Mel‐5, SK‐Mel‐28 and A375 were seeded into 96‐well plates (1.5 × 10^3^ cells per well) and treated with various dosages of lj‐2‐66 (20, 50, 100 and 200 nM) for 24, 48 and 72 h respectively. Then, CCK‐8 assay was used to detect cell viability as described in the methods. The data represent the mean (*n* = 3) ±SD of each group, and an asterisk (*) indicates a significant difference evaluated using one‐way ANOVA (*p* < .05). (b) IC_50_ value was calculated by GraphPad software for 48 h treatment. (c) PIG1 were seeded into 96‐well plates (3 × 10^3^ cells per well) and treated with various dosages of lj‐2‐66 (125, 250, 500 and 1000 nM) for 48 h. Then, CCK‐8 assay was used to detect cell viability as described in the methods. The data represent the mean (*n* = 3) ±SD of each group. IC_50_ value was calculated by GraphPad software. (d) SK‐Mel‐5, SK‐Mel‐28 and A375 were seeded into 6‐well plates (1.5 × 10^3^ cells in each well) and treated with various dosages of lj‐2‐66 (20, 50 and 100 nM) for 48 h. The culture was terminated when clones were visible to the naked eye (about 2–3 weeks). Clones were fixed with 4% paraformaldehyde and stained with 0.5% crystal violet staining solution as described in the methods. The data from multiple experiments are expressed as the mean (*n* = 3) ±SD. Significant differences were evaluated using one‐way ANOVA, and an asterisk (*) indicates a significant difference (*p* < .05)

We designed several 2‐methyl‐7‐chloroquinoline and methylcarbazole hybrid chloroquine derivatives and tested their effect on the viability of Sk‐Mel‐5 cells. As shown in Table 1, the 2‐ethylquinoline and carbazole hybrid compounds effectively inhibited the growth of Sk‐Mel‐5 cells, with IC_50_ values ranging from 130 nM to 11.84 μM. Among the compounds, lj‐2‐66 had the highest activity, with an IC_50_ value of 130 nM. Therefore, we chose lj‐2‐66 for follow‐up experiments to test its anti‐BRAF‐mutant melanoma activity and mechanism. We tested the effect of lj‐2‐66 on the proliferation of melanoma cell lines. lj‐2‐66 inhibited the proliferation of BRAF‐mutant melanoma cells in a dose‐dependent manner (Figures 1a and S2a). The IC_50_ values of lj‐2‐66 in Sk‐Mel‐5, Sk‐Mel‐28 and A375 cells were 130, 80 and 100 nM respectively (Figure 1b). Although chloroquine also suppressed the proliferation of BRAF‐mutant melanoma cells in a dose‐dependent manner (Figure S2b), its IC50 values in Sk‐Mel‐5, Sk‐Mel‐28 and A375 cells were 16, 19 and 7 μM respectively (Figure S2b). The results indicated that lj‐2‐66 had higher antitumour activity than chloroquine in melanoma harboring BRAF mutations. To determine the toxicity of lj‐2‐66 and chloroquine to normal cells, we treated the human melanocyte cell line PIG1 with lj‐2‐66 and chloroquine for 48 h and then evaluated cell viability. The IC_50_ values of lj‐2‐66 and chloroquine in PIG1 cells were 970 and 38 μM respectively (Figures 1c and S2b). The selectivity index (SI) was calculated as the ratio of the IC50 of the drug in PIG1 cells to that in melanoma cells. The SI values of lj‐2‐66 in Sk‐Mel‐5, Sk‐Mel‐28 and A375 cells were 7.5, 12.1 and 9.7 respectively, while those of chloroquine were 2.3, 2.0 and 5.4 respectively. The results indicated that lj‐2‐66 had higher selectivity for BRAF‐mutant melanoma cells than chloroquine. To further examine the effect of lj‐2‐66 on the viability of BRAF‐mutant melanoma cells, we conducted a colony formation assay. Moreover, lj‐2‐66 treatment significantly abrogated BRAF‐mutant melanoma cell colony formation (Figure 1d).

### 3.2 lj‐2‐66 induced G2/M arrest and apoptosis in melanoma cells

**FIGURE 2:**
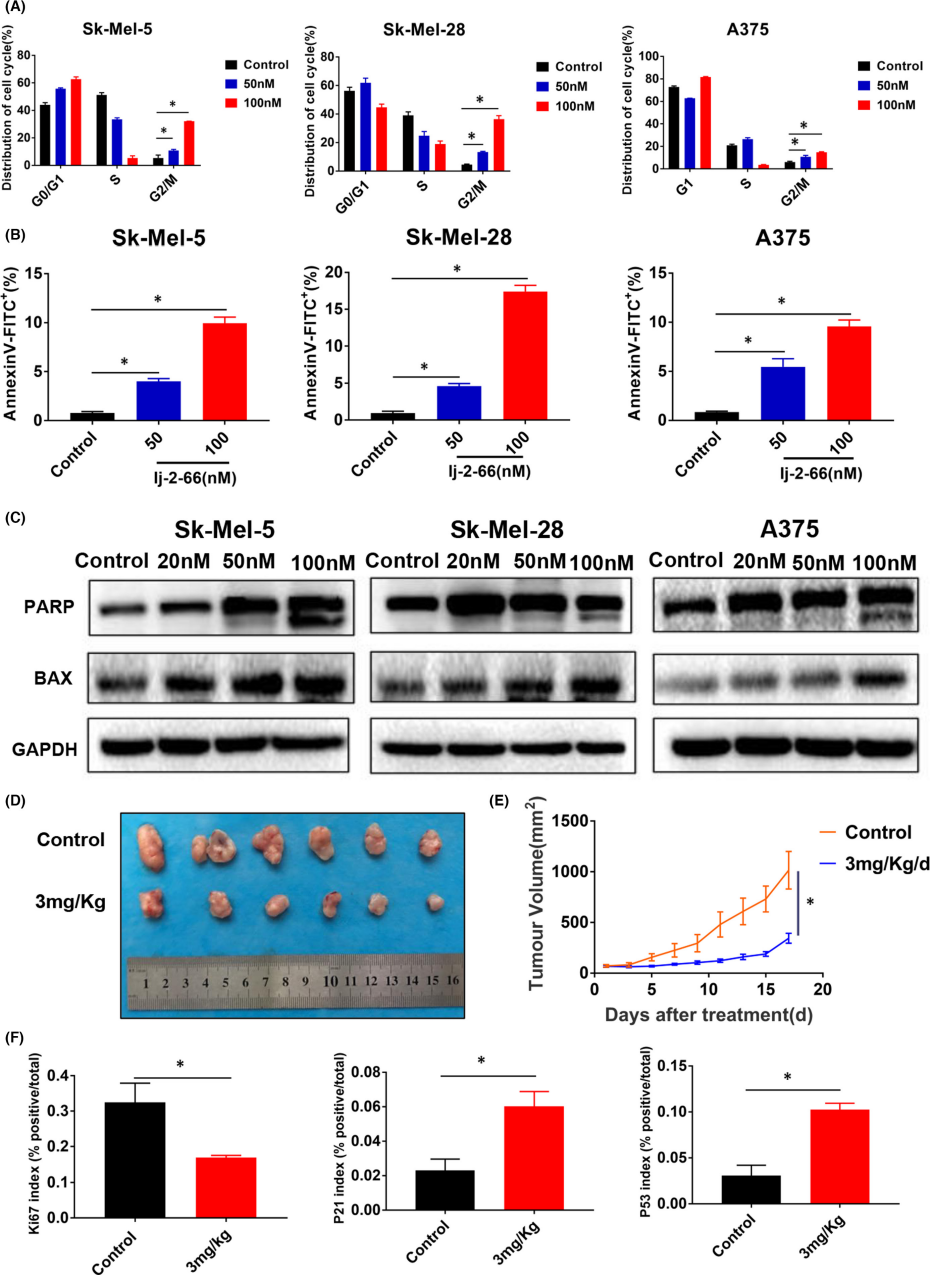
lj‐2‐66 induced G2/M arrest and apoptosis in melanoma cells. (a) SK‐Mel‐5, SK‐Mel‐28 and A375 were seeded into six‐well plates and treated with lj‐2‐66 (50 and 100 nM) for 48 h. Then, cells were fixed with pre‐cooled 70% ethanol and incubated with propidium iodide staining as described in the methods. The data from multiple experiments are expressed as the mean (*n* = 3) ± SD. Significant differences were evaluated using two‐way ANOVA, and an asterisk (*) indicates a significant difference (*p* < .05). (b) SK‐Mel‐5, SK‐Mel‐28 and A375 were seeded into six‐well plates and treated with various dosages of lj‐2‐66 (50 and 100 nM) for 48 h. Then, cells were incubated with Annexin V/propidium iodide staining as described in the methods. The data represent the mean (*n* = 3) ±SD of each group, and an asterisk (*) indicates a significant difference evaluated using one‐way ANOVA (*p* < .05). (c) SK‐Mel‐5, SK‐Mel‐28 and A375 were treated with various dosages of lj‐2‐66 (20, 50, 100 nM) for 48 h, and then, Western blotting was performed for the indicated antibodies. (d) Sk‐Mel‐5 cells (2 × 10^6^) were subcutaneously injected into the right flank of the BALB/c female nude mice (5 weeks old). When the tumuor reached about 50 mm^3^, 3 mg/kg lj‐2‐66 (dissolved in corn oil) or an equal volume of corn oil (Control group) was injected intraperitoneally every day. The pictures were taken after removing the tumuors from mice. (e) Tumour volume was recorded every other day. Data was analysed by GraphPad software. The results are shown as the mean tumour volume ±SD, and an asterisk (*) indicates a significant difference (*p* < .05 student’s *T* test). (f) Ki67, P21 and P53 index (% positive/total). The results represent the means (*n* = 5) ±SD, and asterisk (*) indicates a significant difference (*p* < .05, student’s *t* test)

To explore the possible mechanism of lj‐2‐66’s anti‐BRAF‐mutant melanoma activity effect, we evaluated the cell cycle and apoptosis in melanoma cells treated with 50 and 100 nM lj‐2‐66 for 48 h by flow cytometry. We found that lj‐2‐66 arrested the cell cycle at the G2/M phase boundary and promoted apoptosis (Figure 2a,b).

When treated with 50nM lj‐2‐66, some cells arrested at the G2/M phase boundary, and when the dose increased to 100 nM, the proportion of cells with G2/M arrest increased significantly (Figure 2a). In addition, at a low dose of 50 nM, lj‐2‐66 treatment resulted in apoptosis rates of 4.02%, 4.6% and 5.5% in Sk‐Mel‐5, Sk‐Mel‐28 and A375 cells respectively. At a high dose of 100 nM, the percentages of apoptotic Sk‐Mel‐5, Sk‐Mel‐28 and A375 cells increased to 9.95%, 17% and 9.6% respectively (Figure 2b). To further verify the effect of lj‐2‐66 on apoptosis, we assessed the expression of apoptosis markers. As expected, lj‐2‐66 treatment upregulated BAX expression and induced PARP cleavage (Figure 2c).

### 3.3 lj‐2‐66 suppressed the growth of melanoma in vivo

Our previous results indicated the inhibitory effect of lj‐2‐66 on BRAF‐mutant melanoma cells. To further verify the therapeutic effect of lj‐2‐66 on BRAF‐mutant melanoma in vivo, we used a nude mouse xenograft model as described in the methods section and found that lj‐2‐66 suppressed the growth of BRAF‐mutant melanoma in vivo and did not affect the body weight of the mice (Figures 2d,e and S3a). In addition, Ki67 staining was significantly reduced in the lj‐2‐66 treatment group (Figures S3b and 2f). This result indicated that lj‐2‐66 significantly inhibited the growth of BRAF‐mutant melanoma in vivo.

### 3.4 RNA‐seq analysis identified the effect of lj‐2‐66 on the transcriptome and key pathways

**FIGURE 3:**
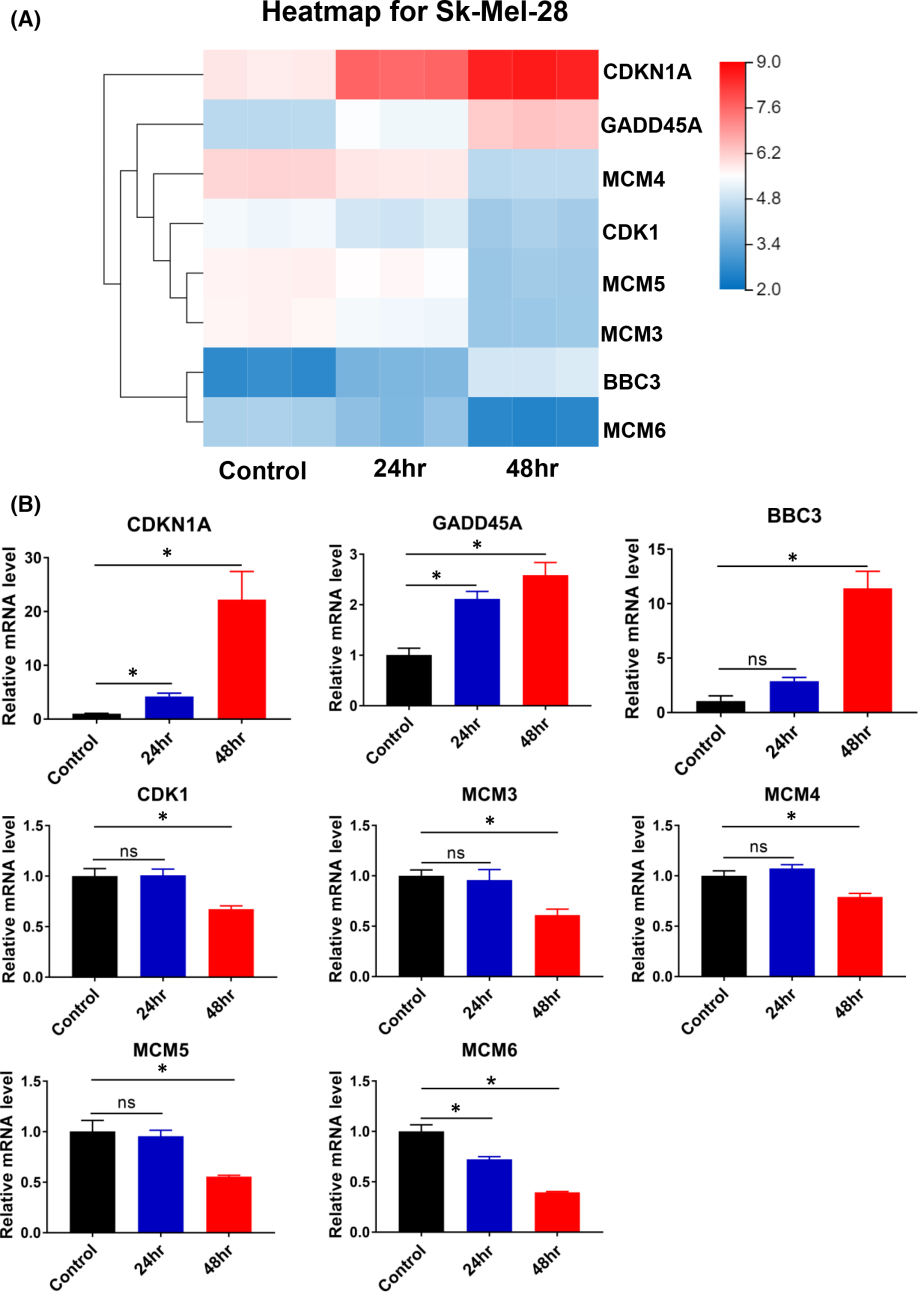
RNA‐seq identified the effect of lj‐2‐66 on the transcriptome. (a) Heatmap showing differentially expressed cell cycle or DNA damage‐related genes in Sk‐Mel‐28 after 100 nM lj‐2‐66 treatment for 24 or 48 h. (b) Sk‐Mel‐28 was treated with lj‐2‐66 at a dosage of 100 nM for 48 h, and then RT‐PCR was performed as described in the methods. The results represent the mean (*n* = 3) ±SD of each group, and an asterisk (*) indicates a significant difference using one‐way ANOVA (*p* < .05)

To further explore the mechanism of lj‐2‐66 in melanoma, we performed RNA‐seq on BRAF‐mutant melanoma cells after lj‐2‐66 treatment and used the Kyoto Encyclopedia of Genes and Genomes (KEGG) database to analyse differentially expressed genes. We found that these genes were involved in multiple pathways, such as cell cycle‐ and tumour‐related pathways (Figure S4). We identified key pathways involved in the cell cycle and DNA damage, consistent with the effect of lj‐2‐66 on G2/M arrest in different melanoma cell lines. Next, we performed RT–PCR to verify the expression levels of genes with the most significant expression changes identified by RNA‐seq, including CDKN1A, GADD45A, BBC3, CDK1, MCM3, MCM4, MCM5 and MCM6 (Figure 3b). These genes play an important role in cell cycle arrest and DNA damage, and their expression changes were consistent with those identified by RNA‐seq (Figure 3a,b).

### 3.5 lj‐2‐66 induced DNA damage by increasing the production of ROS

**FIGURE 4:**
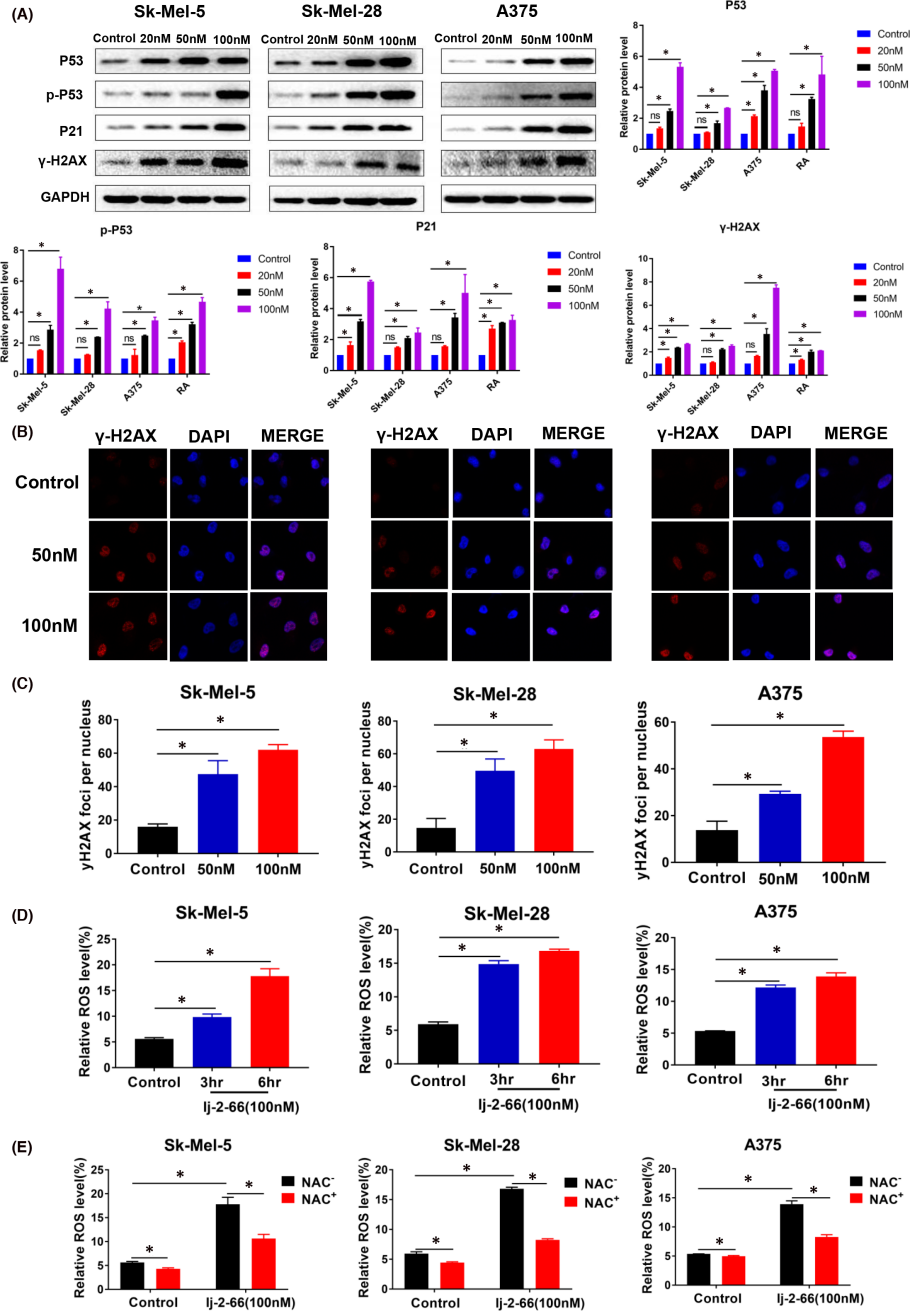
lj‐2‐66 induced DNA damage by increasing the production of ROS. (a) SK‐Mel‐5, SK‐Mel‐28 and A375 were treated with various dosages of lj‐2‐66 (20, 50, 100 nM) for 48 h. And then western blotting was performed for the indicated antibodies. The histograms indicated relative protein expression, as means ±SD, and an asterisk (*) indicates a significant difference using two‐way ANOVA (*p* < .05). (b) SK‐Mel‐5, SK‐Mel‐28 and A375 were seeded on coverslips and exposed to various dosages of lj‐2‐66 (50, 100 nM) for 24 h, and then, immunofluorescence was performed forγ‐H2AX and the photographs were taken by confocal microscope. (c) The data from multiple experiments are expressed as the mean (*n* = 4) ± SD. Significant differences were evaluated using one‐way ANOVA, and an asterisk (*) indicates a significant difference (*p* < .05). (d) SK‐Mel‐5, SK‐Mel‐28 and A375 were treated with 100 nM lj‐2‐66 or equal volume DMSO (control group) for 3 or 6 h and incubated with DCFH‐DA as described in the methods. The results represent the mean (*n* = 3) ±SD of each group, and an asterisk (*) indicates a significant difference compared with control group using one‐way ANOVA (*p* < .05). (e) SK‐Mel‐5, SK‐Mel‐28 and A375 were treated with NAC for 1 hour and subsequently treated with100 nM lj‐2‐66 or equal volume DMSO (control group) for 6 h. Then, the cells were incubated with DCFH‐DA as described in the methods. The data from multiple experiments are expressed as the mean (*n* = 3) ± SD. Significant differences were evaluated using two‐way ANOVA, and an asterisk (*) indicates a significant difference (*p* < .05)

According to previous results, lj‐2‐66 not only caused G2/M arrest but also induced apoptosis. Combining these findings with the RNA‐seq results, we speculated that the anti‐BRAF‐mutant melanoma effect of lj‐2‐66 may be related to the induction of DNA damage. Therefore, we tested the expression levels of the DNA damage marker proteins P53, p‐P53, P21 and γH2AX. As expected, lj‐2‐66 treatment remarkably increased P53, p‐P53, P21 and γH2AX expression in BRAF‐mutant melanoma cells in a dose‐dependent manner (Figure 4a). Moreover, in the lj‐2‐66 treatment group, P21 and P53 staining significantly increased (Figures 2f and S3c,d). Moreover, γH2AX accumulated in the nucleus after treatment with lj‐2‐66 for 24 h (Figure 4b,c). ROS are well recognized as mediators of DNA damage.^19^ Therefore, we tested the effect of lj‐2‐66 on the level of ROS and found that lj‐2‐66 increased the production of ROS in numerous BRAF‐mutant melanoma cell lines (Figure 4d). NAC is recognized as an inhibitor of ROS. We found that the promotive effect of lj‐2‐66 on ROS production was partially suppressed by NAC (Figure 4e). These results suggested that lj‐2‐66 was likely to induce DNA damage by increasing the production of ROS, leading to G2/M arrest and apoptosis.

### 3.6 lj‐2‐66 inhibited the growth of RA

Several clinical trials have shown the antitumour effects of BRAF inhibitors alone or in combination with BRAF and MEK inhibitors against BRAF‐mutant melanoma.^20^, ^21^, ^22^, ^23^, ^24^ However, due to gene mutations (such as BRAF splicing variants, NRAS mutations, MEK1/2 mutations and BRAF amplification), epigenetic and transcriptional changes (such as the downregulation of a series of histone deacetylase (HDAC) genes), and immunological mechanisms (such as a reduction in regulatory T cells (Tregs)), patients treated with BRAF inhibitors (such as vemurafenib) tend to develop drug resistance within 5–7 months.^4^, ^25^, ^26^ Therefore, there is an urgent need to develop new drugs to treat these patients regardless of their BRAFi resistance status.

**FIGURE 5:**
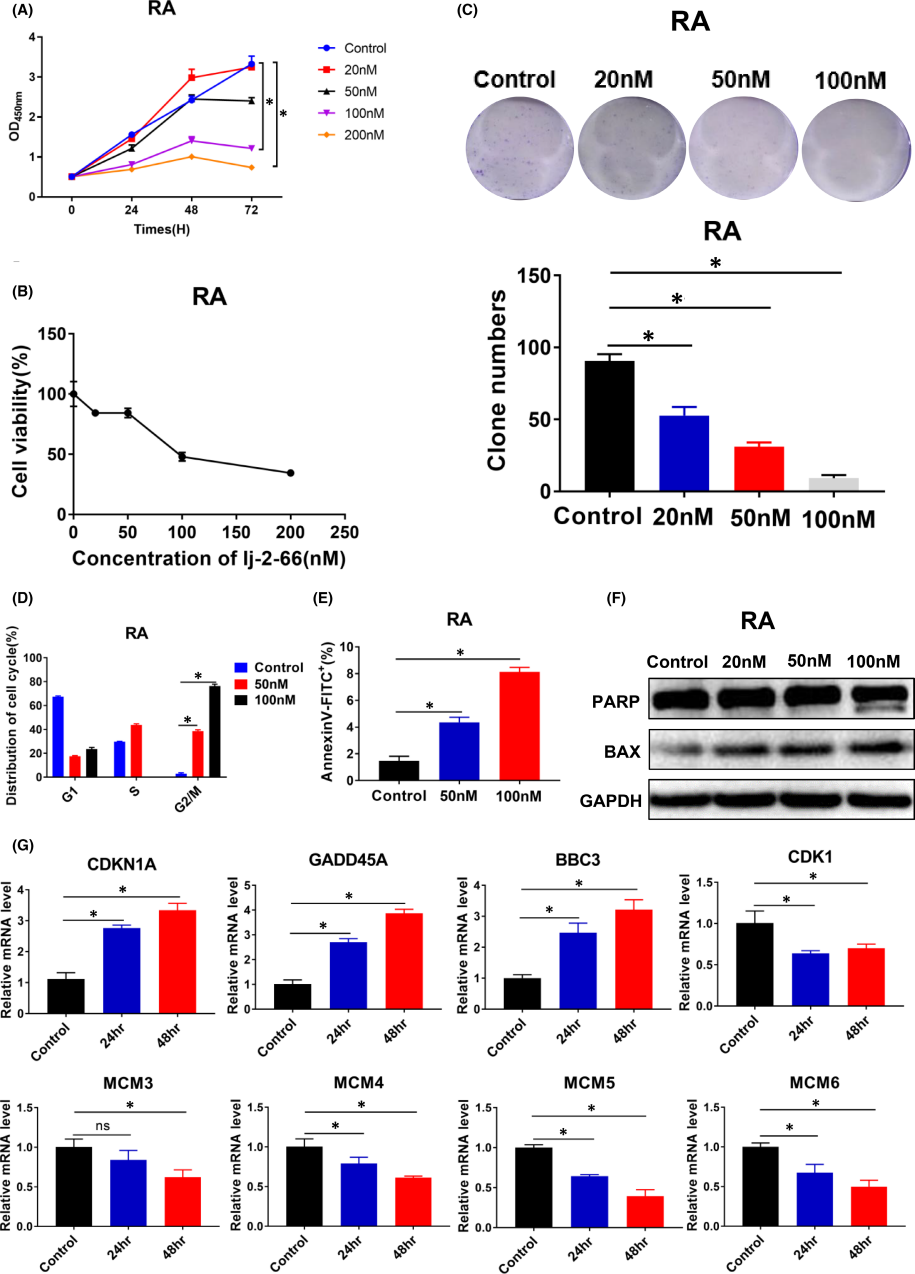
lj‐2‐66 inhibited the growth of RA. (a) RA was seeded into 96‐well plates (1.5 × 10^3^ cells per well) and treated with various dosages of lj‐2‐66 (20, 50, 100 and 200 nM) for 24, 48 and 72 h respectively, and then, CCK‐8 assay was used to detect cell viability as described in the methods. The data from multiple experiments are expressed as the mean (*n* =3) ±SD. Significant differences were evaluated using one‐way ANOVA, and an asterisk (*) indicates a significant difference (*p* < .05). (b) IC_50_ value was calculated by GraphPad software for 48 h treatment. (c) RA was seeded into 6‐well plates (1.5 × 10^3^ cells in each well) and treated with various dosages of lj‐2‐66 (20, 50 and 100 nM) for 48 h. The culture was terminated after clones were visible to the naked eye (about 2–3weeks). The clones were fixed with 4% paraformaldehyde and stained with 0.5% crystal violet staining solution as described in the methods. The results represent the mean (*n* = 3) ± SD of each group, and an asterisk (*) indicates a significant difference using one‐way ANOVA (*p* < .05). (d) RA was seeded into six‐well plates (3 × 10^5^ per well) and treated with various dosages of lj‐2‐66 (50 and 100 nM) for 48 h. Then, cells were fixed with precooled 70% ethanol and incubated with propidium iodide staining as described in the methods. The results represent the mean (*n* = 3) ±SD of each group, and an asterisk (*) indicates a significant difference using two‐way ANOVA (*p* < .05). (e) RA was seeded into six‐well plates (3 × 10^5^ per well) and treated with various dosages of lj‐2‐66 (50 and 100 nM) for 48 h. Then, cells were incubated with Annexin V/propidium iodide staining as described in the methods. The results represent the mean (*n* = 3) ±SD of each group, and an asterisk (*) indicates a significant difference using one‐way ANOVA (*p* < .05). (f) RA was treated with various dosages of lj‐2‐66 (20, 50, 100 nM) for 48 h, and then, Western blotting was performed for the indicated antibodies. (g) RA was treated with lj‐2‐66 at a dosage of 100 nM for 48 h, and then, RT‐PCR was performed as described in the methods. The results represent the mean (*n* = 3) ±SD of each group, and an asterisk (*) indicates a significant difference using one‐way ANOVA (*p* < .05)

**FIGURE 6:**
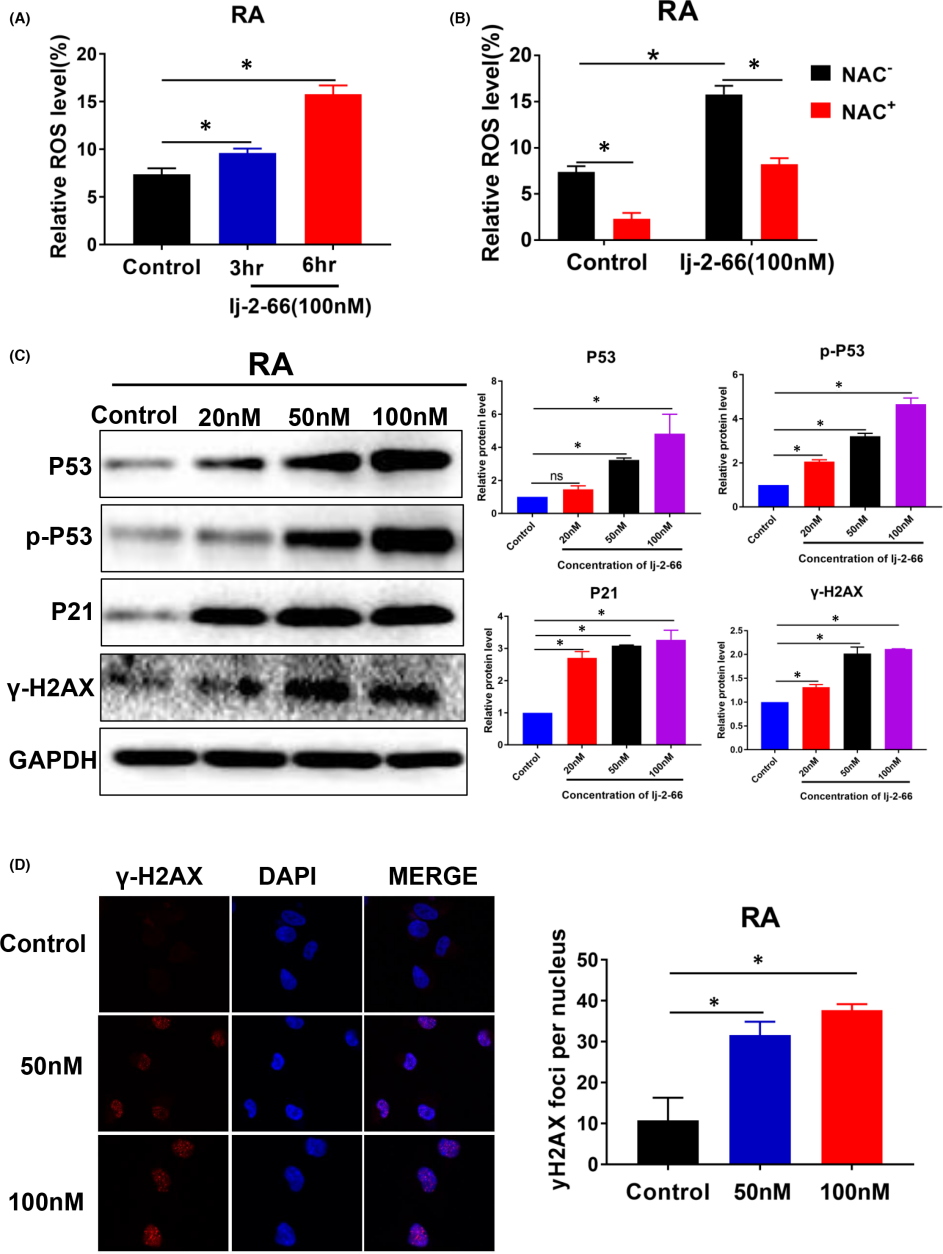
lj‐2‐66 induced DNA damage by increasing the production of ROS in RA. (a) RA was treated with 100 nM lj‐2‐66 or equal volume DMSO (control group) for 3 or 6 h, then incubated with DCFH‐DA as described in the methods. The results represent the mean (*n* = 3) ±SD of each group, and an asterisk (*) indicates a significant difference using one‐way ANOVA (*p* < .05). (b) RA was treated with NAC for 1 h and subsequently treated with 100 nM lj‐2‐66 or equal volume DMSO (control group) for 6 h, then incubated with DCFH‐DA as described in the methods. The results represent the mean (*n* = 3) ±SD of each group, and an asterisk (*) indicates a significant difference using two‐way ANOVA (*p* < .05). (c) RA was treated with various dosages of lj‐2‐66 (20, 50 and 100 nM) for 48 h. Then, Western blotting was performed for the indicated antibodies. The histograms indicated relative protein expression, as means ± SD, and an asterisk (*) indicates a significant difference using two‐way ANOVA (*p* < .05). (d) RA was seeded on coverslips (2 × 10^4^ /well) and exposed to various dosages of lj‐2‐66 (50 and 100 nM) for 24 h, and then, immunofluorescence was performed forγ‐H2AX and the photographs were taken by confocal microscope. The results represent the mean (*n* = 4) ± SD of each group, and an asterisk (*) indicates a significant difference using one‐way ANOVA (*p* < .05)

We tested the effect of lj‐2‐66 on BRAFi‐resistant RA melanoma cells and found that lj‐2‐66 inhibited the growth of RA cells in a dose‐dependent manner with an IC50 of 139nM after treatment for 48 h (Figure 5a,b). In addition, lj‐2‐66 reduced colony formation in a dose‐dependent manner (Figure 5c). Next, we tested the effect of lj‐2‐66 on the cell cycle and apoptosis in RA cells. The results suggested that lj‐2‐66 arrested the cell cycle at the G2/M phase boundary and induced apoptosis. At 50nM, lj‐2‐66 induced G2/M arrest in RA cells, and at 10 nM, lj‐2‐66 induced a significant increase in G2/M arrest in RA cells (Figure 5d). Moreover, at a dose of 50 nM, lj‐2‐66 treatment resulted in an apoptosis rate of 4.35%, and when the dose was raised to 100 nM, the rate increased to 8.13% (Figure 5e). Therefore, we detected the expression of apoptosis‐related proteins in RA cells treated with lj‐2‐66. As expected, lj‐2‐66 treatment increased the expression of BAX and caused PARP cleavage (Figure 5f). We also performed RT–PCR to verify the expression of CDKN1A, GADD45A, BBC3, CDK1, MCM3, MCM4, MCM5 and MCM6. As expected, the changes in the expression of these genes were consistent with those identified by RNA‐seq (Figure 5g). In addition, lj‐2‐66 treatment increased the ROS level, and this increase was partially suppressed by NAC (Figure 6a,b). We also tested the expression of DNA damage‐related proteins and found that lj‐2‐66 increased the expression of P53, p‐P53, P21 and γH2AX in a dose‐dependent manner (Figure 6c). Moreover, we observed accumulation of γH2AX in the nucleus (Figure 6d).

## 4 DISCUSSION

Chloroquine is regarded as a promising drug for tumour treatment, but it usually requires high doses, which may cause extensive and serious side effects. Therefore, the development of chloroquine derivatives with higher anticancer activity has become a hot topic in cancer treatment.^10^, ^11^, ^12^, ^13^, ^14^ In this study, we designed several chloroquine derivatives—2‐methyl‐7‐chloroquinoline and methylcarbazole hybrid compounds that have certain BRAF‐mutant melanoma inhibitory activities—and identified the most active compound, lj‐2‐66 (Table 1).

The IC_50_ values of lj‐2‐66 in Sk‐Mel‐5, Sk‐Mel‐28 and A375 cells were 130, 80 and 100 nM respectively (Figure 1b). In addition, it had similar suppressive effects on RA cells and non‐BRAFi‐resistant melanoma cells, independent of the BRAFi resistance status (Figure 5a,b). Furthermore, we found that the antimelanoma activity of lj‐2‐66 was achieved via the induction of G2/M arrest and apoptosis (Figure 2a,b). Then, RNA‐seq was performed to explore the mechanism of lj‐2‐66’s effect on BRAF‐mutant melanoma cells. The results showed that cell cycle‐ and tumour‐related pathways were significantly altered after lj‐2‐66 treatment (Figure S4). Then, we verified the genes with the most significant expression changes identified by RT–PCR and found that the expression of CDKN1A, GADD45A and BBC3 was upregulated and that the expression of CDK1, MCM3, MCM4, MCM5 and MCM6 was downregulated after lj‐2‐66 treatment (Figure 3b).

CDKN1A, also called p21, mediates p53‐dependent cell cycle arrest.^27^, ^28^ At a high level, p21 inhibits the function of cyclin‐dependent kinases (CDKs), including the cyclin D/CDK4/6 complex and cyclin E/CDK2 complex, leading to cell cycle arrest.^29^ The GADD45A protein is localized in the nucleus and interacts with cdc2/cyclinB1 kinase to inhibit progression through the G2/M phase boundary in the cell cycle.^30^ In addition, GADD45A is involved in DNA damage, apoptosis, cell damage and other growth regulation processes.^31^, ^32^ BBC3, also called PUMA, is a downstream molecule of p53 that can induce DNA damage and promote apoptosis by increasing ROS levels.^33^ The CDK1 protein is a key cyclin that accelerates the G2/M transition and decreases the entire cell cycle time.^34^ The expression of MCM protein in several malignant tissues (such as breast, gastrointestinal, lung and ovarian cancers) is higher than that in normal tissues.^35^, ^36^ Evidence shows that abnormal expression of MCM promotes the occurrence and development of tumours through a variety of mechanisms.^36^ All of these genes are related to the cell cycle or DNA damage.

DNA damage, that is, the chemical or physical changes in DNA in cells, can affect the interpretation and transmission of genetic information.^19^ When DNA damage occurs, a network of events is activated, namely the DNA damage response (DDR), which includes checkpoint activation, DNA damage recognition, cell cycle arrest and apoptosis.^37^, ^38^ Among these events, cell cycle arrest, which can prevent continued division of DNA‐damaged cells, is one of the most important aspects of the DNA damage response. Combining these observations with the transcriptome sequencing results, we speculated that lj‐2‐66 can suppress melanoma by inducing DNA damage. Therefore, we evaluated the expression levels of DNA damage‐related proteins and found that lj‐2‐66 increased the expression levels of P‐53, p‐P53, P21 and γH2AX in a dose‐dependent manner (Figure 4a). In addition, we found that γH2AX accumulated in the nucleus after treatment with lj‐2‐66 (Figure 4b,c). p53 is a widely studied tumour suppressor that affects the response of various cells to DNA damage.^39^, ^40^ In addition, P53 can be a target of ROS.^41^ Histone H2AX is a chromatin factor that has been widely studied in the DDR.^42^ These results indicate that lj‐2‐66 induces DNA damage.

ROS, which consist of a series of short‐lived molecules such as •OH, H2O2 and O2‐, mediate DNA damage. Studies have shown that ROS can promote apoptosis and G2/M arrest by inducing DNA damage.^19^, ^43^, ^44^ To verify whether the DNA damage‐inducing effect of lj‐2‐66 is mediated by ROS, we measured the level of ROS in melanoma cells treated with lj‐2‐66 by flow cytometry. The results showed that lj‐2‐66 increased the level of ROS, and this effect was partially inhibited by NAC (Figure 4d,e). This finding indicates that lj‐2‐66 may exert an anti‐BRAF‐mutant melanoma effect through the induction of DNA damage by increasing the ROS level. Similar to the effect of lj‐2‐66 in melanoma cells, it has been demonstrated that glucocorticoid receptor‐mediated signalling exerts a significant impact on breast cancer cells, increasing intracellular levels of ROS and DNA damage and negatively affecting repair processes. Therefore, synthetic glucocorticoids may be coadministered with lj‐2‐66 to boost the potential therapeutic efficacy of the latter for melanoma harboring BRAF mutations.^45^, ^46^

Although we found that lj‐2‐66 has a much higher IC_50_ value in immortalized nontumorigenic cells than in melanoma cells, there are still limitations of this study because we were not able to calculate the therapeutic index of the compound. In future studies, to provide more evidence supporting final clinical administration, the safety of the compound in vivo needs to be tested by approaches such as drug toxicology, metabolism and other related studies.

## 5 CONCLUSIONS

**FIGURE 7:**
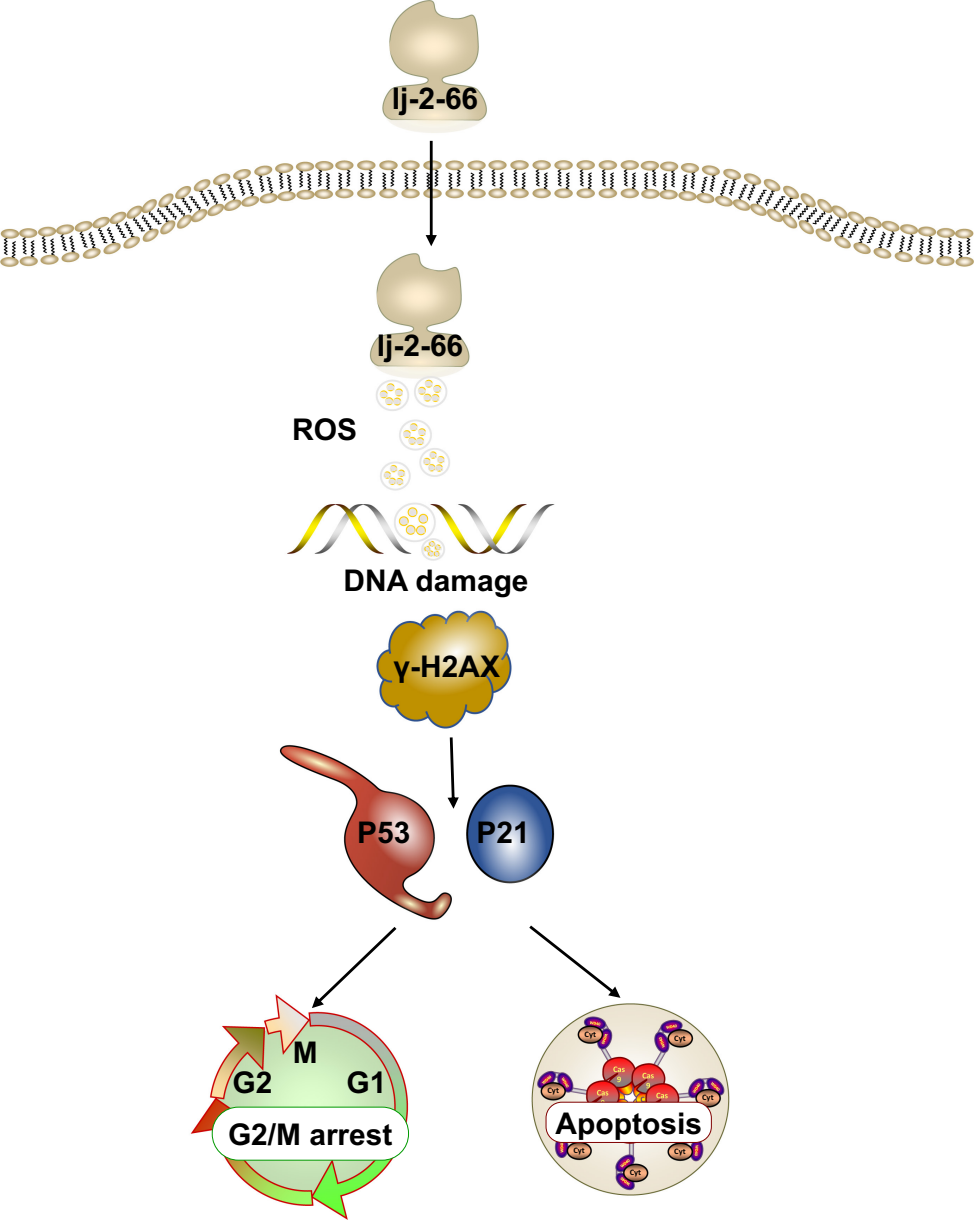
Schematic diagram of the mechanism of action of lj‐2‐66. lj‐2‐66 induces DNA damage by increasing the levels of ROS, leading to G2/M phase arrest and apoptosis of melanoma cells, and subsequently inhibited the growth of BRAF‐mutant melanoma

In summary, we synthesized the chloroquine derivative lj‐2‐66, which can induce DNA damage by increasing the level of ROS, leading to G2/M arrest and apoptosis in melanoma cells and subsequently inhibiting the growth of melanoma both in vivo and in vitro. These results provided evidence that the compound lj‐2‐66 has an anti‐BRAF‐mutant melanoma effect (Figure 7).

## CONFLICT OF INTEREST

The authors confirm that there are no conflicts of interest.

## AUTHOR CONTRIBUTIONS

**Cong Peng:** Conceptualization (lead). **Jiaoduan Li:** Methodology (equal); Writing – original draft (lead). **Jing Long:** Methodology (equal). **Jianglin Zhang:** Conceptualization (equal); Funding acquisition (equal). **Nian Liu:** Methodology (equal). **Bei Yan:** Methodology (equal). **Ling Tang:** Methodology (supporting). **Xiang Chen:** Funding acquisition (equal).

## Supporting information

fig S1‐S4

## Data Availability

The datasets generated and/or analysed during the current study are available from the corresponding author on request.
