# Supplementary material for: Novel chloroquine derivative suppresses melanoma cell growth by DNA damage through increasing ROS levels
Source: J Cell Mol Med. 2022 Mar 25;26(9):2579–93. doi: 10.1111/jcmm.17260 (PMC9077290; doi:10.1111/jcmm.17260)
Supplement: Supplementary file 1 — fig S1‐S4 [file JCMM-26-2579-s001.docx]

**Fig S1**

**Synthesis of 2-methyl-7-chloroquinoline and methylcarbazole hybrid compounds.** To a solution of 1 (888 mg, 5 mmol), 2 (1.16 g, 20 mmol) and TMSN3 (1.15 g, 10 mmol) in benzene (30 mL), phenyliodine bis(trifluroacetate) (4.3 g, 10 mmol) was added portion wise in a 5-10 minutes period at room temperature. After stirring the reaction mixture for 24 h at room temperature, Et3N (12.5 mL) was added and then stirred for 10 min. The solvents were removed under reduced pressure, following column chromatography the pure product 3 (806 mg, 69%) was obtained. 1H NMR (500 MHz, CDCl3) δ 8.23 (d, J = 8.9 Hz, 1H), 8.07 (s, 1H), 7.52 (d, J = 9.0 Hz, 1H), 7.46 (s, 1H), 3.06 (q, J = 7.0 Hz, 2H), 2.80 (s, 3H), 1.30 (t, J = 7.1 Hz, 3H); HRMS found: 234.0687.

To a solution of 3 (58 mg, 0.25 mmol) in 3.1 mL EtOH was added NaOH (50 mg, 1.25 mmol). After stirring for 5 min, 4 (63 mg, 0.3 mmol) was added in one portion. After reaction completed, the mixture was extracted with EtOAc, and the combined organic layers were then washed with saturated brine, dried over anhydrous Na2SO4, and concentrated in vacuo to afford the crude products, which was purified by column chromatography with petroleum/ethyl acetate (7:3) to give lj-2-64 (87 mg, 82%). 1H NMR (500 MHz, CDCl3) δ 8.17-8.12 (m, 2H), 8.09 (d, J = 7.7 Hz, 1H), 7.78 (d, J = 8.8 Hz, 1H), 7.58-7.51 (m, 2H), 7.48-7.37 (m, 4H), 7.33-7.26 (m, 3H), 3.88 (s, 3H), 2.82 (s, 3H), 2.53 (s, 3H); 13C NMR (125 MHz, CDCl3) δ 198.2, 159.6, 148.9, 148.7, 146.8, 141.6, 141.5, 135.8, 134.6, 128.6, 128.2, 127.5, 126.7, 126.5, 125.9, 123.3, 123.1, 122.6, 122.3, 120.5, 120.2, 119.9, 108.9, 108.6, 29.3, 25.5, 13.1; HRMS found: 425.1426.

To a solution of 1 (139 mg, 0.785 mmol), 4 (657 mg, 3.14 mmol) and TMSN3 (181 mg, 1.57 mmol) in benzene (1.5 mL), phenyliodine bis(trifluroacetate) (675 mg, 1.57 mmol) was added portion wise in a 5-10 minutes period at room temperature. After stirring the reaction mixture for 24 h at room temperature, Et3N (0.5 mL) was added and then stirred for 10 min. The solvents were removed under reduced pressure, following column chromatography the pure product lj-2-65 (170 mg, 56%) was obtained. 1H NMR (500 MHz, CDCl3) δ 8.56 (s, 1H), 8.13 (s, 1H), 8.05 (d, J = 7.8 Hz, 1H), 8.00 (d, J = 8.6 Hz, 1H), 7.75 (d, J = 8.9 Hz, 1H), 7.55 (t, J = 7.6 Hz, 1H), 7.48-7.41 (m, 2H), 7.40-7.36 (m, 2H), 7.30 (t, J = 7.6 Hz, 1H), 3.91 (s, 3H), 2.81 (s, 3H); 13C NMR (125 MHz, CDCl3) δ 195.1, 159.7, 148.7, 146.1, 144.4, 141.8, 135.8, 128.3, 128.2, 127.9, 127.4, 127.0, 126.8, 124.0, 122.91, 122.87, 122.1, 120.8, 120.5, 120.4, 109.2, 108.5, 29.4, 25.5; HRMS found: 385.1099, 387.1075

Sodium borohydride (15 mg, 0.397 mmol) was added in portions to a solution of lj-2-65 (56 mg, 0.145 mmol) in MeOH (1.2 mL) under cooling in an ice bath. The mixture was stirred for 3.5 h and the solvent subsequently removed under reduced pressure. The residue was taken up in H2O and extracted with EtOAc. The combined organic layers were dried over Na2SO4, filtered, and concentrated under reduced pressure. The residue was purified by column chromatography to give 48 mg (85%) of lj-2-66. 1H NMR (500 MHz, DMSO) δ 8.23 (s, 1H), 8.16 (d, J = 9.1 Hz, 1H), 8.12 (d, J = 7.7 Hz, 1H), 7.94 (d, J = 1.7 Hz, 1H), 7.79 (s, 1H), 7.55 (d, J = 8.2 Hz, 1H), 7.49 (d, J = 8.5 Hz, 1H), 7.47-7.41 (m, 3H), 7.18 (t, J = 7.4 Hz, 1H), 6.55 (s, 1H), 6.30 (s, 1H), 3.82 (s, 3H), 2.74 (s, 3H); 13C NMR (125 MHz, DMSO) δ 160.7, 151.2, 148.6, 141.4, 140.4, 134.5, 133.8, 127.8, 127.1, 126.2, 125.6, 122.9, 122.3, 122.2, 120.7, 120.1, 119.4, 119.2, 109.6, 109.4, 71.9, 29.4, 25.6; HRMS found: 387.1270, 389.1236.

A solution of lj-2-66 (18 mg, 0.047mmol) and CDI (10 mg, 0.060 mmol) in anhydrous MeCN (2 mL) was refluxed for 24 h. Concentrated under vacuum and preparative thin-layer chromatography afforded pure product lj-2-67 (19 mg, 95%). 1H NMR (500 MHz, CDCl3) δ 8.09 (s, 1H), 7.99 (d, J = 7.8 Hz, 1H), 7.88 (s, 1H), 7.67 (d, J = 9.0 Hz, 1H), 7.52 (t, J = 7.6 Hz, 1H), 7.49-7.39 (m, 3H), 7.34-7.22 (m, 5H), 7.19 (s, 1H), 6.90 (s, 1H), 6.64 (s, 1H), 3.88 (s, 3H), 2.72 (s, 3H); 13C NMR (125 MHz, CDCl3) δ 160.6, 148.8, 145.8, 141.5, 141.1, 137.6, 135.4, 129.8, 128.6, 127.3, 126.9, 126.6, 126.1, 124.6, 123.4, 122.3, 122.1, 120.8, 120.5, 120.2, 119.7, 119.5, 109.3, 108.8, 62.1, 29.2, 25.7; HRMS found: 437.1524, 439.1504.

Ph3PCH3Br (56 mg) was added to a flame-dried round-bottom flask, evacuated, backfilled with N2 three times, and suspended in THF (1 mL). To this vigorously stirring heterogeneous solution was added NaOtBu (15 mg), and the reaction was allowed to stirred at room temperature for 15 min until a bright yellow heterogeneous mixture was achieved. The resulting solution was cooled to 0 °C, and lj-2-65 (50 mg, 0.13 mmol) was added slowly Upon complete addition, the cooling bath was removed, and the reaction was allowed to stir for 24 h before filtering through Celite and concentrating. The crude material was purified by silica gel column chromatography to give the pure product lj-2-68 (36 mg, 72%). 1H NMR (500 MHz, CDCl3) δ 8.08 (s, 1H), 8.04-7.97 (m, 2H), 7.72 (d, J = 8.9 Hz, 1H), 7.50 (t, J = 7.5 Hz, 1H), 7.43-7.38 (m, 2H), 7.36-7.29 (m, 2H), 7.25-7.20 (m, 2H), 6.07 (s, 1H), 5.40 (s, 1H), 3.86 (s, 3H), 2.81 (s, 3H); 13C NMR (125 MHz, CDCl3) δ 160.2, 149.4, 148.8, 146.6, 141.5, 140.9, 135.0, 131.0, 127.9, 127.6, 126.5, 126.1, 124.7, 124.1, 123.0, 122.8, 122.7, 120.4, 119.2, 118.6, 115.4, 108.7, 108.5, 29.2, 25.4; HRMS found: 383.1306, 385.1289.

A mixture of 6 (216 mg, 1 mmol) and NaN3 (204 mg, 3.1 mmol) in DMF (1.2 mL) was stirred at 95−100 °C for 20 h then coevaporated with toluene−H2O azeotrope in vacuo. The residue was stirred with H2O, filtered and dried well. Flash chromatography affords 7 (181 mg, 83%) as faint brown solid. 1H NMR (500 MHz, CDCl3) δ 7.99 (d, J = 1.8 Hz, 1H), 7.96 (d, J = 8.9 Hz, 1H), 7.46-7.42 (m, 1H), 7.04 (s, 1H), 2.75 (s, 3H); 13C NMR (125 MHz, CDCl3) δ 160.3, 149.0, 145.9, 136.3, 127.3, 126.3, 123.3, 118.1, 109.1, 25.4.

7 (22 mg, 0.1 mmol), 8 (21 mg, 0.1 mmol) and sodium L-ascorbate (2 mg, 0.01 mmol) were dissolved in THF (3 mL) under N2 flow in a flame dried Schlenk flask and added to a mixture of triethylamine (TEA) (0.2 mL) as a ligand. The flask was flushed with N2, which was followed by the addition of CuSO4 (0.8 mg, 0.005 mmol). The mixture was stirred at refluxing for 5 h. The THF was removed under vacuum and the mixture was washed with water followed by PE and EA to give lj-2-114 (26 mg, 62%) as a brown solid. 1H NMR (500 MHz, CDCl3) δ 8.71 (s, 1H), 8.29 (s, 1H), 8.19-8.16 (m, 2H), 8.09-8.05 (m, 2H), 7.58-7.49 (m, 4H), 7.46 (d, J = 8.1 Hz, 1H), 7.32-7.29 (m, 1H), 3.91 (s, 3H), 2.86 (s, 3H); 13C NMR (125 MHz, CDCl3) δ 160.8, 149.9, 149.6, 141.5, 141.2, 141.2, 136.8, 128.4, 128.3, 126.3, 124.5, 123.9, 123.3, 122.7, 120.5, 120.4, 120.3, 119.4, 119.1, 118.1, 116.8, 109.0, 108.8, 29.3, 25.5.

**Fig S2**


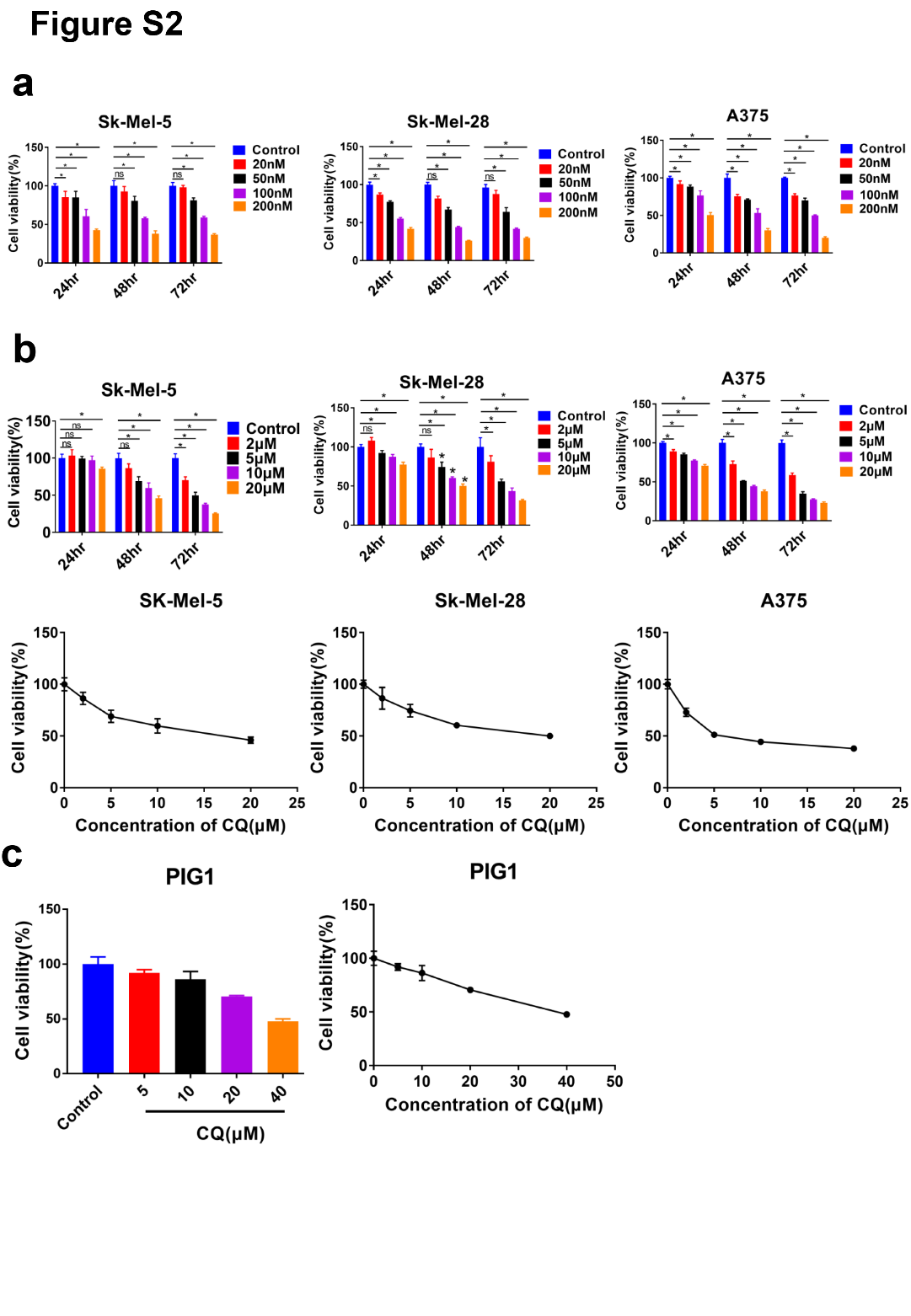


**Effects of chloroquine on the proliferation of melanoma cells. (a)** SK-Mel-5, SK-Mel-28 and A375 were seeded into 96-well plates (1.5x10^3^ cells per well) and treated with various dosages of lj-2-66 (20nM, 50nM, 100nM, 200nM) for 24hr, 48hr and 72hr, respectively. Then CCK-8 assay was used to detect cell viability as described in the methods. The data represent the mean (n=3) ± SD of each group, and an asterisk (*) indicates a significant difference evaluated using two-way ANOVA (p < 0.05). **(b)** SK-Mel-5 and SK-Mel-28 were seeded into 96-well plates (1.5x10^3^ cells per well) and treated with various dosages of chloroquine (2uM, 5uM, 10uM, 20uM) for 24hr, 48hr and 72hr, respectively. Then CCK-8 assay was used to detect cell viability as described in the methods. The data from multiple experiments are expressed as the mean (n = 3) ± S.D. Significant differences were evaluated using two-way ANOVA, and an asterisk (*) indicates a significant difference (p < 0.05). IC_50_ value was calculated by GraphPad software for 48hr treatment.

**Fig S3**


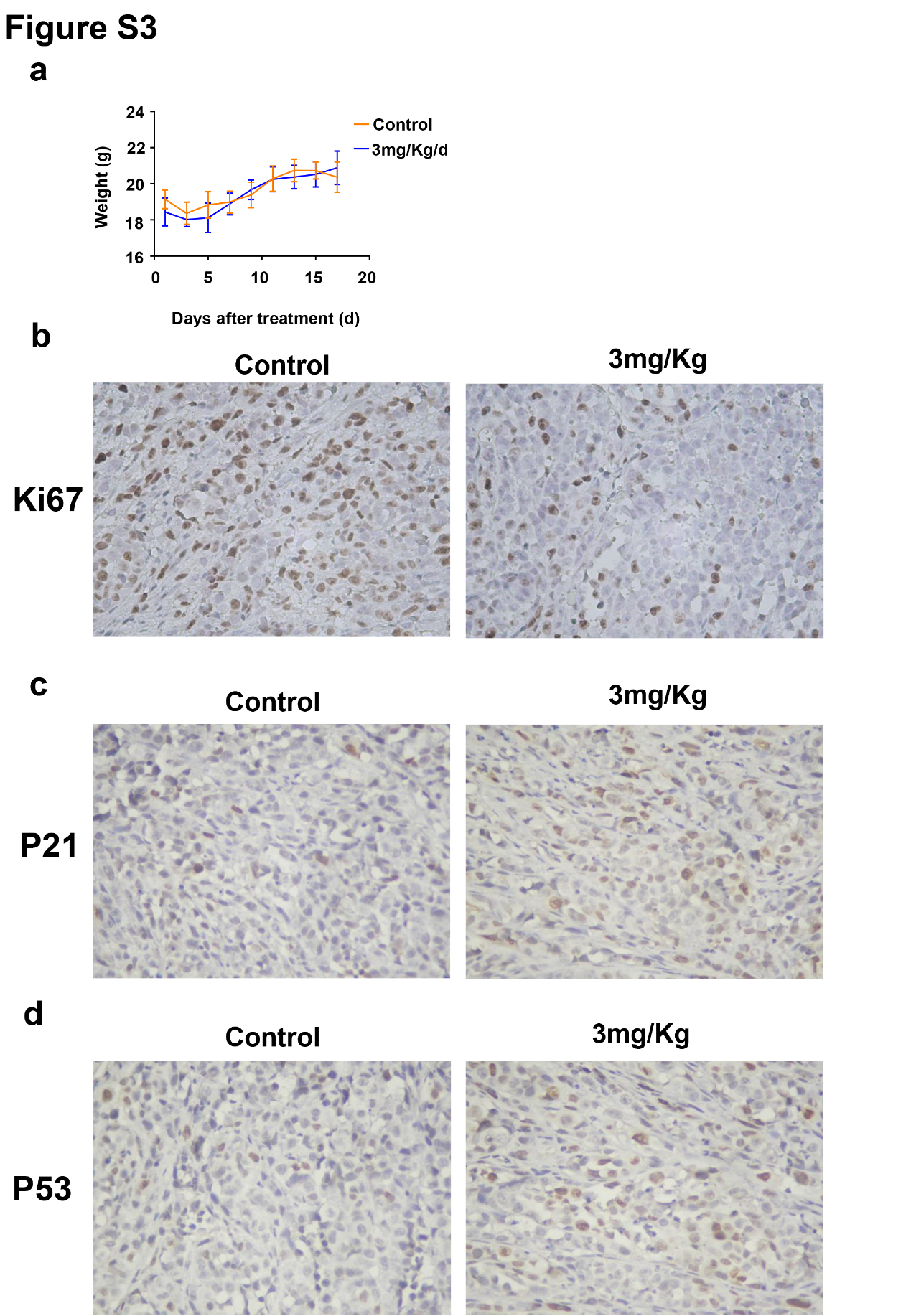


**lj-2-66 suppresses xenograft tumor growth in vivo. (a)** Mice weight was recorded every other day. Data was analyzed by GraphPad software. The results are shown as the mean mice weight± SD, and there isn’t a significant difference (p > 0.05 one-way ANOVA). **(b-d)** Representative images of IHC staining of Ki67, P21 and P53 in tumor tissues.

**Fig S4**


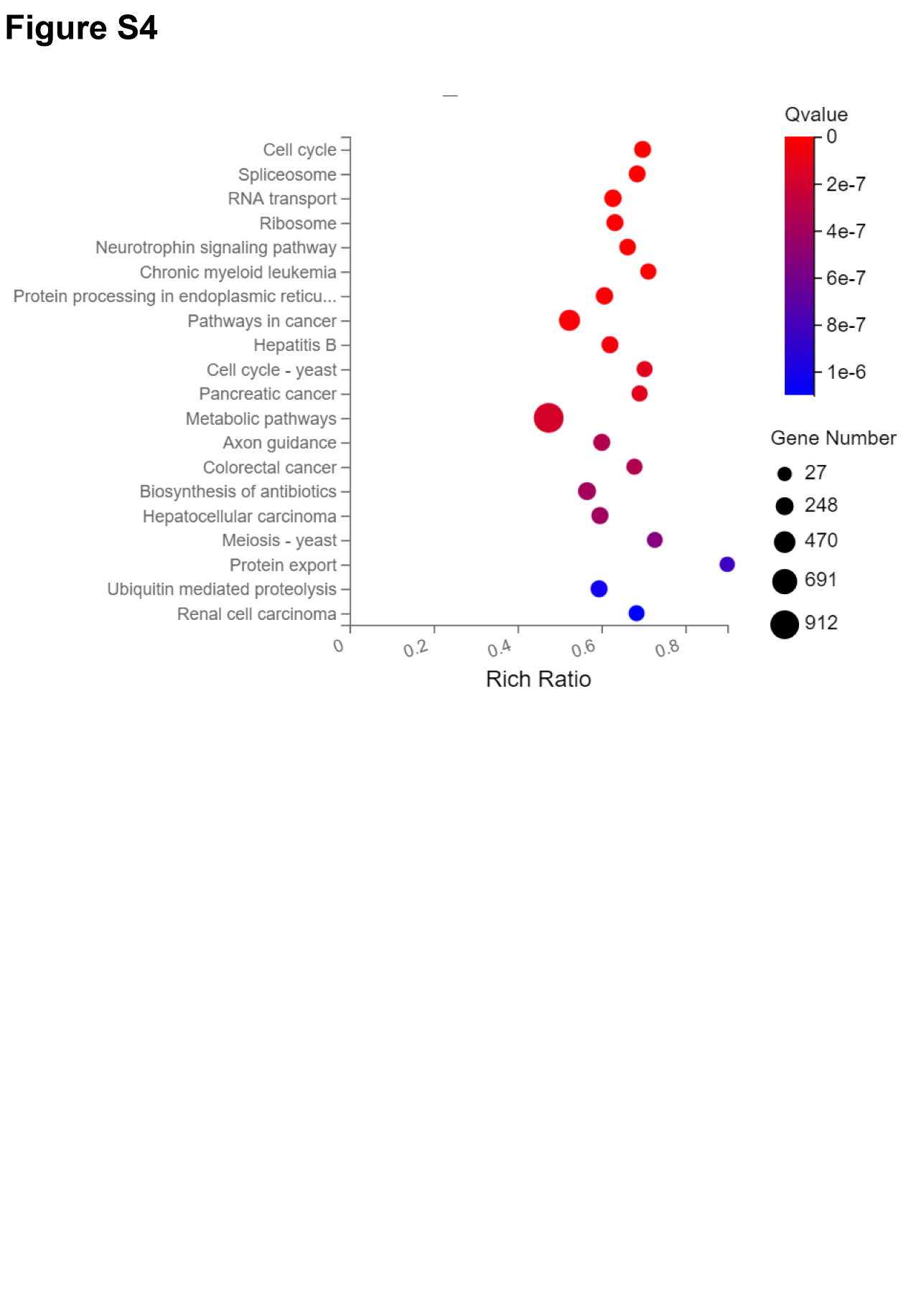


**Top 20 enriched KEGG pathways after lj-2-66 treatment.** The KEGG pathway was used to analyze the pathways related to the differential expression genes between control group and 100nM lj-2-66 treatment for 48hr. The top 20 positively enriched pathways are shown in the bubble chart. The x-axis is the enrichment score, and the y-axis is the enriched pathways.
